# Behavioral Patterns in Special Education. Good Teaching Practices

**DOI:** 10.3389/fpsyg.2017.00631

**Published:** 2017-05-02

**Authors:** Manuela Rodríguez-Dorta, África Borges

**Affiliations:** Departamento de Psicología Clínica, Psicobiología y Metodología, Universidad de La LagunaCanary Islands, Spain

**Keywords:** diversity, educational needs, special education, good teaching practices, behavioral patterns

## Abstract

Providing quality education means to respond to the diversity in the classroom. The teacher is a key figure in responding to the various educational needs presented by students. Specifically, special education professionals are of great importance as they are the ones who lend their support to regular classroom teachers and offer specialized educational assistance to students who require it. Therefore, special education is different from what takes place in the regular classroom, demanding greater commitment by the teacher. There are certain behaviors, considered good teaching practices, which teachers have always been connected with to achieve good teaching and good learning. To ensure that these teachers are carrying out their educational work properly it is necessary to evaluate. This means having appropriate instruments. The Observational Protocol for Teaching Functions in Primary School and Special Education (PROFUNDO-EPE, v.3., in Spanish) allows to capture behaviors from these professionals and behavioral patterns that correspond to good teaching practices. This study evaluates the behavior of two special education teachers who work with students from different educational stages and educational needs. It reveals that the analyzed teachers adapt their behavior according the needs and characteristics of their students to the students responding more adequately to the needs presented by the students and showing good teaching practices. The patterns obtained indicate that they offer support, help and clear guidelines to perform the tasks. They motivate them toward learning by providing positive feedback and they check that students have properly assimilated the contents through questions or non-verbal supervision. Also, they provide a safe and reliable climate for learning.

## Introduction

Diversity in education is a reality that appears before the term “attention to diversity” was coined. In every classroom, there are students who have diverse educational needs arising from different aspects. Covering them means including all educational resources to take advantage of the characteristics of these students and enhance their learning (Zavala and de la Torre, 2015).

In education, good teaching practices have always been related to what should be done to provide adequate education to achieve good learning (Del Valle Ballón, 2012). Some definitions relate good teaching practices to examples of conduct and successful procedures (Anna, 2003 is cited Cid-Sabucedo et al., 2009) or with activity that has been developed, has been evaluated and has been successful (Cid-Sabucedo et al., 2009).

Most of the extraordinary educational measures undertaken to address these schools imply an individualized programming, supports and personal and material resources. The teacher must be supported by specialists such as teachers of Therapeutic Pedagogy (TP) (Gómez Montes, 2005). This is a discipline designed to correct development or learning dysfunctions. It is aimed at those students with temporary or permanent disability that makes more difficult for them to learn the lessons taught, or requires specialized attention to achieve the maximum level of education according to their possibilities (Decree 157/1986 of October 24 on the Management of Therapeutic Pedagogy in an Integrated System, 1986).

In the Canary Islands, students who require very specific attention and resources are cared for in the Specialized Unit (*Aulas Enclave*, in Spanish) which are located in regular centers of compulsory education (Alegre, 2000).

Teachers specialized in special education require specific competence. They must acquire this competence in their initial (Pegalajar-Palomino, 2014) and permanent education (Sykes et al., 2010; Conklin, 2012; Pegalajar-Palomino, 2014). To make their work in the classroom effective they must use, mix and adapt different strategies, resources and opportunities to the different characteristics, levels and needs of students (Ruiz Rodríguez, 2003; Martínez Geijo, 2007; Pegalajar-Palomino, 2011; González-Peiteado, 2013). Therefore, Special Education is different from the one that takes place in the regular classroom, demanding more dedication from the teacher.

The concept of good teaching practices is becoming more popular and starting to be used at a theoretical level (Bain, 2006), although it has not been defined or pointed which teaching conducts or behaviors represents this concept. The first research studies on regard this topic have been developed with university teachers (Díaz et al., 2015; Borges et al., 2016a,c). However, there is a large body of research about the effectiveness of teachers and teaching. They highlight specific aspects that are of interest, although most are not contextualized in Special Education.

Thus, teaching planning and the organization of the classroom seem to be one of the aspects of most influence the performance in different educational levels (Brophy and Good, 1986). Related to this aspect, several authors point out the importance and influence of an appropriate structure of the lessons to help students in their cognitive development and improve their performance (Mortimore et al., 1988; Renkl and Helmke, 1992; Brown, 2009; Hunt et al., 2009; Orlich et al., 2010).

With regard to the transmission of content by the teacher, research highlights the importance of clearly explaining the objectives of each lesson (Melton, 1978; Cotton, 1995). Other authors point out other aspects which are also relevant, such as the presentation of information in an organized way, pointing out transitions to new topics, using a variety of examples and frequent reminder of the essential principles (Maddox and Hoole, 1975; Smith and Cotton, 1980; Kallison, 1986; Mayer and Gallini, 1990; Hiebert et al., 1991).

Other studies indicate aspects are related with student’s motivation. This is based on psychoeducational principles from which arise various strategies related to arouse and maintain attention, generate cognitive dissonance and positive expectations for learning (Hernández, 1991; Hernández and García, 1995 is cited Hernández-Jorge, 2005). Strategies such as individualized attention, reinforcement, frequent supervision, public and private praises, etc. (Murillo et al., 2011) and the use of diverse activities to adapt to different moments, circumstances, students, etc. (Dalton, 2007; Hunt et al., 2009) are the best for the student to learn. Another important aspect is the use of activities that require the active participation of students. In this sense, the traditional “learning by doing” strategy is still effective (Muijs and Reynolds, 2001).

With respect to the climate generated in class and that is given by the interaction between teacher and students, it is important that the students feel comfortable to participate in the activities (Muijs and Reynolds, 2001). Simple aspects such as greeting or asking about general aspects of the students at the beginning of the class make students feel safe and comfortable (Hernández-Jorge, 2005).

Also, research refers to aspects related to the classroom’s discipline. It has been found that if teachers apply an adequate control method to the unwanted behaviors of the students in the classroom, they will obtain the maximum benefit from teaching (Omoteso and Semudara, 2011). In this sense, the most successful teachers in the control of their class are those who, among other aspects, adequately analyze the different disturbing stimuli of the classroom, use simple rules and make explicit to students and use clear behavioral indicators both verbal and non-verbal (Hernández-Jorge, 2005).

Another aspect that is highlighted by research and that favors a quality education is the evaluation and continuous monitoring of the students (Daloz, 1986; Stronge et al., 2004; Killen, 2005; Brookhart, 2009; Orlich et al., 2010; Murillo et al., 2011). Evaluation is relevant as a way to identify whether students are getting or not proper results (Muijs and Reynolds, 2001; Anderson, 2004; Hattie and Timperley, 2007; Murillo et al., 2011). To do this, the teacher can ask questions or observe the work the student is performing (Pellicer and Anderson, 1995). However, the assessment should not stop here, but the teacher must motivate the students with feedback during their learning process (Muijs and Reynolds, 2001; Anderson, 2004; Hattie and Timperley, 2007; Murillo et al., 2011; Pegalajar-Palomino, 2011), which is also a way to motivate them.

With respect to the orientation that the teacher offers students in their learning process, research emphasizes it is important that the teacher solves any doubt that arises in the students to facilitate the adequate understanding of new concepts (Berliner, 1983; Anderson, 1989, 2004). Provide support (“Scaffolds”) to students so that they can carry out the activities (Palincsar and Brown, 1984; Van de Grift, 2007) is important. Also, the research notes that, among other things, presenting new material in small steps, providing clear and detailed instructions (Rosenshine, 1979), asking questions and providing guided practice has been proved effective, especially with younger students and those who have lower academic abilities than the rest of their age group (Muijs and Reynolds, 2003; Houtveen et al., 2004; Houtveen and Van de Grift, 2006).

Additionally, it is important to highlight it is essential to take into consideration the diversity of needs and abilities within a classroom in order to ensure an education of good quality. This means answering the different needs, expectations, abilities, characteristics, motivations, cultures, previous knowledge, learning rhythms, etc., present in the students (Murillo et al., 2011). Also, to consider activities that are challenging for all students is important (Houtveen et al., 1999; Ainscow et al., 2001; Ainscow, 2007).

To verify that these aspects are taking place is important to evaluate the educational work of the teacher in the classroom. For this reason it is essential to have instruments that are not only accurate, valid and reliable, but it is also important that the instruments are sensitive and allow capturing the differential behavior of teachers in their attention to students with different characteristics and needs. The assessment tools used to operationalize the quality of teachers to measure their teaching performance are various, such as teacher’s materials, briefcases, questionnaires and surveys, interviews and observation (Jiménez, 1999; García and Congosto, 2000; Rodríguez and Ibarra, 2013).

The classroom observation is a proper procedure because it implies a good way of knowing the reality of the classroom, thus allowing us to dig deeper into the exchanges that occur within it every day (Mayorga and López, 2005), and provide evidence to identify areas that should be improved (Pianta and Hamre, 2009).

The objective of this study is to analyze the behavior of two teachers from Special Education in their interaction with students from Preschool and Primary Education who present different educational needs. The aim is to determine whether there are differences on their teaching practices depending on the students with who they interact and their needs. In the first place, we assume that the behavior of Special Education teachers during their educational work in the classroom is adjusted to the characteristics of students with special educational needs. Secondly, we assume that to the extent that the behavior of these teachers is in line with the characteristics of their students, this is indicative of good teaching practices.

## Materials and Methods

### Methodology and Design

For the determination of behavioral patterns in the classroom, observational methodology has been used, whose design, taking into account the axes referred to units of study, temporality of registration and dimensionality (Anguera et al., 2001), is nomothetic, monitoring, and multidimensional.

### Participants

The participants in this study have been selected intentionally. Given the institutional nature of this research, we contacted the Canary Islands Government Education Department to request authorization for collecting the information. They proposed two schools to us. Each school proposed a teacher of Special Education. These two teachers teach children of different educational stages. The first one works in TP, he is 52 years old and 22 years of experience. The second teacher works in Specialized Unit, he is 53 years old and 18 years of experience.

In order to collect the continuous flow of the behaviors that take place in the classroom, it is necessary to analyze the students’ behaviors. For this reason, students’ are studied in their interaction with the teacher. This research involves a total of 11 students of different educational stages that present specific educational needs and require a different educational response. The teacher of TP attends both students of Preschool Education and students of Primary School, while the teacher in the Specialized Unit works only students of Preschool Education. **Table 1** shows the number of students corresponding to each teacher, the educational stage in which they are and their ages.

**Table 1:** Participating students per teacher.

Teacher	Students	Educational stage	Age of students
Therapeutic Pedagogy	1	Preschool Education	5–6 years


	3	Primary School	8–9 years
Specialized Unit (*Aula Enclave*)	7	Preschool Education	5–6 years

Three observers participated in the coding of the videos. Two of them are students of Psychology and one has a Master in Educational Psychology.

### Instruments

#### Observational Protocol for Teaching Functions in Primary School and Special Education (PROFUNDO-EPE, v.3., in Spanish) (Rodríguez-Dorta, 2015)

The observational instrument was based on the Protocol of Teaching Functions (PROFUNDO v2, in Spanish) (Rodríguez-Naveiras, 2011), created for the observation of teaching functions in an extracurricular program of psychoeducational intervention. PROFUNDO v2 is based on the model of teaching functions of Hernández-Jorge (2005).

The PROFUNDO v.2 has been adapted for the study of the educational functions in different educational stages: to university education, obtaining the Protocol of Observation of Teaching Functions in University (PROFUNDO-UNI, v.2, in Spanish) (Díaz, 2014) and Primary and Special Education, obtaining the instrument that is presented below, Protocol of Observation of Teaching Functions in Primary and Special Education (PROFUNDO-EPE, v.3, in Spanish) (Rodríguez-Dorta, 2015; **Table 2**).

**Table 2:** Observational protocol in the Teaching Functions in Primary School and Special Education (PROFUNDO-EPE, v.3., in Spanish).

MACROCATEGORIES Teacher’s Functions	CODES	
1. Organization	Teacher’s Organization	TO
2. Teacher’s Communicability	Teacher’s Explanation	TE
3. Motivation	Reinforcement	RF
	Motivation	MO
4. Behavioral Control	Control	CL
5. Guidance and Advice	Guidance	GU
	Non-verbal Revision	NR
6. Interaction	General Interactions	GI
	Use of the Diary	SD
7. Evaluation	Verbal Revision	VR
8. Students’ Interactions	Students Participation	SP
	Answer the Teacher	AT
	Classroom Disruption	CD
	General Interactions	GI
Instrumental Categories	Other Behaviors	X
	Unobservable	Y
	Teacher Leaves the Classroom	Z

The PROFUNDO-EPE, v.3 it is based on the Teaching Functions Model and analyzes those functions which can be directly observed: (a) organization function: planning of education and control over the context; (b) teacher’s communicability function: teacher’s ability to communicate the contents so that they are understood by students; (c) motivation function: teacher’s ability to encourage students to learning; (d) behavior control function: group regulation, order and discipline; (e) orientation and advice function: guide students in their learning; (f) interaction function: teacher–student relationship to generate motivation, correct mistakes, and expand the information is working; (g) evaluation function: propose criteria to check whether the learning objectives have been achieved and whether the teaching process has been adequately performed.

The ***Organization Function*** is included in the code *Teacher’s Organization* (TO). This code gathers all the tasks that the teacher develops in the classroom and has an organizational component (organization of teaching material, planning and structuring of the class and organization of students to work).

The ***Teacher’s Communicability Function*** is collected through the code *Teacher’s Explanation* (TE). This code represents the theoretical expositions of the teacher.

The ***Motivation Function*** is composed by two codes. The *Reinforcement* (RF) code formed by verbalizations or non-verbal teacher behaviors aimed at positively reinforcing students’ behavior. The *Motivation* (MO) code represents teacher verbalizations that allow students to choose on some aspect of the task, verbalizations aimed at generating motivation toward the task or activity, verbalizations that highlight some aspect of the task to be performed or the anticipation of reward.

The ***Behavior Control Function*** is included in the *Control* (CL) code. This code is formed by all the teaching behaviors of criticism, threat, or punishment directed to the students.

The ***Orientation and Advice Function*** is included in the *Guidance* (GU) and *Non-verbal Revision* (NR) codes. The first one contains the indications, questions, clues and corrections directed to the students during the accomplishment of an activity. Also, collect the teacher’s answers to questions raised by the students regarding what is being worked in class. The second includes the silent supervision of the activity or task of the students by the teacher.

The ***Interaction Function*** includes two codes. The *General Interactions* (GI) code formed by the verbalizations of the teacher directed to the students that are not related to the activity, task or theoretical content that is being worked. The code *Use of the Diary* (SD) formed by the behaviors of the teacher consisting of use the student’s diary to communicate with their families.

Finally, the ***Evaluation Function*** is composed by the *Verbal Revision* (VR) code. This code consists of all those questions of the teacher directed to check if the students have acquired the contents or aspects worked.

The observation protocol also includes an eighth category to observe the student behavior when they interact with the teacher. Also, the protocol includes a last ***instrument category*** to complete the constant flow of the instructors’ behavior and which collects other types of behavior which are not connected to the typified behaviors.

Thus, the category ***Interventions of the Students*** has four codes. Two of them include the positive interventions of the students. *Students Participation* (SP) formed by the interventions of the students on their own initiative directed to the teacher to present some idea or opinion, ask a question, etc. related to what is being worked on class. *Answer the Teacher* (AT) formed by the students’ answers (verbally or through behavior) to a question, approach, comment, indication, etc. of the teacher referring to what is being worked on class. Negative interventions are listed in the *Classroom Disruption* (CD) code. This code consists of verbal or non-verbal behaviors of the students who are against the logical norms of the classroom (hitting or insulting a classmate, getting up and making noise in the middle of the class, breaking up material, etc.) that interrupt the rhythm of the class and call the attention of the teacher to apply a control. The GI code includes the neutral interventions of the students. This code represents students’ verbalizations addressed to the teacher that are not related to the activity or content that is being worked on class.

The ***Instrumental category*** includes three codes. The code *Other Behaviors* (X) that is formed by those behaviors of the teacher that do not correspond with the teaching functions (to speak with a person who enters the classroom, to speak by the mobile, etc.). The code *Unobservable* (Y) formed by those moments in which it is impossible to observe or codify the behavior of the teacher. The code *Teacher Leaves the Classroom* (Z) that collects those moments in which the teacher leaves the classroom.

As it was mentioned before, this observational instrument is part of the instruments of evaluation of teacher behavior, having been cross-validated. The PROFUNDO v2 (Rodríguez-Naveiras, 2011) has been applied to evaluate instructors of extracurricular programs for students with high ability in Canary Islands and two Mexican States. The PROFUNDO-UNI v2 (Díaz, 2014) was applied to teachers in Canary Islands and Mexico.

#### Operationalization of Good Teaching Practices in Special Education

Given the particular characteristics of teaching Special Education, the PROFUNDO-EPE, v.3 is used to extract behavior patterns that correspond to good teaching practices in this area (**Table 3**), which are explained below.

**Table 3:** Behavioral patterns considered good teaching practices.

Criteria Conduct	Consequent Conducts
Teacher’s Organization (TO)	- Motivation (MO)

Teacher’s Explanation (TE)	- Teacher’s Organization (TO)
	- Motivation (MO)
	- Verbal Revision (VR)

Guidance (GU)	- Motivation (MO)
	- Non-verbal Revision (NR)
	- Verbal Revision (VR)

Non-verbal Revision (NR)	- Reinforcement (RF)
	- Guidance (GU)

Students Participation (SP)	- Reinforcement (RF)
	- Guidance (GU)
	- Non-verbal Revision (NR)
	- Verbal Revision (VR)

Answer the Teacher (AT)	- Reinforcement (RF)
	- Guidance (GU)
	- Non-verbal Revision (NR)
	- Verbal Revision (VR)

Classroom Disruption (CD)	- Control (CL)

General Interactions (GI) of teacher	- General Interactions (GI) of students

General Interactions (GI) of students	- General Interactions (GI) of teacher

After arranging the necessary materials for class or giving action guidelines to students to begin working (TO), it is important to motivate them by presenting, for example, the activity as something attractive or entertaining (MO). When the teacher presents the content in a theoretical way (TE) it is appropriate to continue to give guidance to students, organizing them to work and implement what has been explained (TO). The teacher should also encourage the students to work (MO), or try to check whether students have assimilated adequately the content that was just explained (VR). Also, it is a good teaching practice when after assistances or instructions on how to perform a task (GU), the teacher encourages the students to work (MO), supervises in silence their work (NR) or checks that students have understood the instructions (VR). When the teacher is supervising in silence the work of the students (NR), it is appropriate in this context to give them positive feedback when they are doing their job correctly (RF), or to provide them with aid or instruction when they are not doing activities properly (GU).

When there are positive interventions of students, either on their own initiative (SP) or in response to the teacher (AT), good practices that teachers apply are positive feedback (RF), orient the students (GU), or supervise their interventions in silence (NR), or through questions (VR).

Also, it is appropriate that teachers apply a negative contingency (CL) after a disruptive behavior of the student (CD).

Finally, the GI of the teacher followed by the GI of the students or vice versa are good teaching practices. On the one hand, it contributes to the generation of a safe and reliable climate. On the other, it represents a contrast that facilitates the student’s moments of disconnection, allowing them to return to the task with a higher level of attention.

#### Instruments of Registration and Coding

The teacher’s behaviors are registered with two different video cameras: JVC and Sony.

For the coding process we used software of the Augenv.δ program for the evaluation of behaviors (Montero and Montero, 2012, unpublished).

### Procedure

First, we obtained the signed informed consent from the student’s parents and of from the teachers to record them. After, we proceeded to film 20 class hours for each teacher in the period between January and February 2011. The recording of the 20 h was carried out over several weeks being framed within the same quarter. This allowed us to collect a sufficient sample of teacher behavior in their educational work in the classroom.

In the TP classroom the students attended certain days, the rest is developed in the regular classroom with the corresponding teacher. The TP teacher attends Preschool Education students in a few days and Primary School students on other days. Therefore, we proceeded to record the days in which this teacher taught classes to the student of Preschool Education and the days in which he imparted classes to the students of Primary School. We recorded the entire period of the sessions.

In the Specialized Unit, the teacher attends students throughout all day. These students require special support in all or most of the areas or subjects of the curriculum. The space of the where the Specialized Unit is located has special conditions. It’s not ordinary classroom, it has different spaces (kitchen, adapted bathroom, work room, mathematical room, and psychomotricity room). This place is adapted to the needs of the students. We couldn’t record in all the space of this classroom; therefore we did into on consecutive days and only when the students were in the work room or in the math room.

For the selection of the sessions, two criteria were taken into account: we deleted the first 2 h of recording of each teacher to avoid the reactivity bias and selected those sessions where the teachers could be easily observed, so that their coding could be carried out without difficulty.

Observers signed a confidentiality agreement and were trained following a standardized procedure (Rodríguez-Naveiras, 2011; Cadenas et al., 2012; Díaz, 2014; Rodríguez-Dorta, 2015).

We determine the number of sessions to code through optimization study of the Theory of Generalizability (TG) (Blanco-Villaseñor, 1991; Blanco et al., 2000, 2010). In the case of the teacher of Therapeutic Pedagogy, since he attends students from both Preschool Education and of Primary School, we did the optimization for these two situations. However, we incorporated more sessions of the optimization study identifies as necessary to ensure stability of the behavioral patterns of teacher (Rodríguez-Dorta and Borges, 2015b).

The length of the sessions which were selected for the coding process was established using GT. A period of time is consider optimal when the generalizability coefficient achieves a value higher than 0.90. A total of four sessions of 25 min each were coded from the teacher of TP when he was interacting with students from Preschool. Three sessions of 20 min each were coded in the case of the students from Primary school. For the teacher of Specialized Unit, four sessions of 10 min each were coded.

### Data Analysis

The analysis of inter-observer reliability was conducted through the Cohen’s Kappa Coefficient (Cohen, 1960, 1968), using the program of statistical analysis SPSS v.15 and through the Generalizability Theory (GT; Cronbach et al., 1972), using the programs EduG 6.0 and SAGT v.1.0.

To obtain behavioral patterns we used the sequential lag analysis through the program GSEQ v.5.1. (Bakeman and Quera, 1996), based in determining if one conduct follows another with a higher probability than the one expected randomly. We take a previous conduct or criteria from which we count the times that other (consequent) conducts follow it immediately after, with a first lag and after two conducts in a second, giving a positive or excitatory dependency when the value of Z is higher than 1,96. To determine the extent of the association between behaviors we have calculated the coefficient Yule’s Q (Yule and Kendal, 1957 is cited Lloyd et al., 2013) through the program GSEQ v.5.1.

### Optimization Study

The observational methodology is very costly in time and resources, which makes necessary to have valid, accurate and reliable instruments and procedures in place to implement it efficiently. In this sense, the Decision Study of the GT (Blanco-Villaseñor, 1991; Blanco and Anguera, 2000; Blanco et al., 2000, 2010) is especially useful because it allows to determine what the minimum of sessions and time to code are (Borges and Rodríguez-Dorta, 2015; Rodríguez-Dorta, 2015; Rodríguez-Dorta and Borges, 2015a,b).

Because this process involves the generalization of the same behaviors collected from a context and particular circumstances, this work was carried out for each of the contexts studied. First, the optimization session time is performed and secondly the number of sessions.

Optimizing the number of sessions to encode is usual in observational methodology. However, when the sessions are long-lasting, it is also important to optimize the time of encoding sessions. Since the GT works with discrete variables and time is a continuous variable, we have taken as units of time variable sections of 5 min, choosing three consecutive sections for each context. Next, a trained observer encoded these time sections (Borges and Rodríguez-Dorta, 2015; Rodríguez-Dorta, 2015; Rodríguez-Dorta and Borges, 2015a). Then, we performed a decision study of the time sections. The results allow to conclude that the duration of the session is relatively short in the three contexts, with a maximum of 25 min (**Table 4**).

**Table 4:** Time sections optimization.

Unifaceta Crossover Design TxC. Random Estimation Plan. Measurement Plan C/T.
**Teacher**	**Coef. G Rel.**	**Coef. G Abs.**	**Number of sections**
Therapeutic Pedagogy Preschool	0.912	0.906	5 (25 min)
Therapeutic Pedagogy Primary	0.916	0.914	4 (20 min)
Specialized Unit (*Aula Enclave*)	0.956	0.953	2 (10 min)

Afterward, we set the number of sessions to encode. We selected two sessions with the optimal duration of each context. A trained observer performed the coding of these sessions. Later, we made a decision study for the sessions. The results show that two sessions are needed in all contexts (**Table 5**).

**Table 5:** Optimization of the sessions.

Unifaceta Crossover Design TxC. Random Estimation Plan. Measurement Plan C/T.
**Teacher**	**Coef. G Rel.**	**Coef. G Abs.**	**Number of sessions**
Therapeutic Pedagogy Preschool	0.970	0.970	2
Therapeutic Pedagogy Primary	0.901	0.901	2
Specialized Unit (*Aula Enclave*)	0.983	0.981	2

To check whether the contribution of the decision study is sufficient, we began codifying the number of sessions set for each context and we continued to include sessions on the saturation criterion. In order to do this, we stopped to encode sessions when new relevant behavioral patterns do not appear with the addition of sessions. This procedure allows us to ensure stability of the behavioral patterns in the contexts studied (Rodríguez-Dorta and Borges, 2015b).

We found that coding time established by the decision study is not enough because significant new patterns take place with the inclusion of sessions. Therefore, for TP with students of Primary School we included one more session of the decision study identified as necessary, while for TP with students of Preschool Education and for Specialized Unit it was necessary to include two more sessions.

### Inter-observer Reliability

Observation requires monitoring. So, following the criterion of Patterson (1982), the reliability was calculated at 20% of the encoded session (a session from each context: TP with students of Primary School, TP with students of Preschool Education and Specialized Unit with students of Preschool Education). We calculated the reliability of each observer with expert observer, through Kappa and TG, and among all with TG. In order to avoid bias, the observers were unaware of when reliability would be calculated. Values ranged between 0.83 and 1 for the Kappa index and 0.92 and 0.98, in TG, being suitable all indices obtained (Fleiss, 1981; Bakeman and Gottman, 1986; Hintze and Matthews, 2004).

## Results

### Analysis of Behavioral Patterns

The differences on the behavior of teachers based on the context in which they perform their educational work are evident through the behavioral patterns that take place. Then, significant excitatory patterns in the first and second lag for each criterion behavior in each educational context were shown. The residual value of the consequent conducts is in parentheses and the value of Q of Yule is in the next column. Those patterns that were considered good teaching practices are bolded.

When the criterion behavior is TO, this conduct is followed by TE or AT in Therapeutic Education with students of Primary School in the first lag. In Specialized Unit with students of Preschool Education, TO is followed by interventions of students on their own initiative (*Student Participation*, SP) or responses to the question posed by the teacher (AT) at first lag. In the second lag, it is followed by TO in both teachers (**Table 6**).

**Table 6:** Organization function.

Teacher	Criteria Conduct	Consequent Conducts			
		Lag 1		Lag 2	
		Codes	Yule’s Q	Codes	Yule’s Q
Therapeutic Pedagogy	TO	–		TO (9.10)	0.82
Preschool					
Therapeutic Pedagogy		**TE** (4.28)	0,68	**TO** (5.38)	0.56
Primary		AT (2.85)	0,33		
Specialized Unit (*Aula Enclave*)		SP (2.35)	0,33	TO (4.77)	0.53
		AT (2.68)	0,34		

*TO, Teacher’s Organization; TE, Teacher’s Explanation; SP, Student Participation; AT, Answer the Teacher*.

When the code TE was taken as a criterion behavior (see **Table 7**), meaningful patterns were obtained in TP with students of Preschool Education. In the first lag, this behavior is followed by interventions of students on their own initiative (SP) or a verbal verification by the teacher (VR). It is appropriate that teachers try to check that students understand and properly acquire the content they are working. In the second lag, TE is followed by aids or instructions of teacher (GU) or by students responses to the question posed by the teacher (AT).

**Table 7:** Teacher’s Communicability Function.

Teacher	Criteria Conduct	Consequent Conducts			
		Lag 1		Lag 2	
		Codes	Yule’s Q	Codes	Yule’s Q
Therapeutic Pedagogy	TE	**SP** (9.04)	0.81	**GU** (3.95)	0.49
Preschool		**VR** (5.57)	0.61	AT (3.61)	0.44

*TE, Teacher’s Explanation; SP, Student Participation; VR, Verbal Review; GU, Guidance; AT, Answer the Teacher*.

In **Table 8**, the results when the behavior criterion is RF are presented, finding meaningful patterns in TP with students of Preschool Education and in Specialized Unit with student Preschool Education. In the first case, RF is followed by TE or VR, in first lag. In the second case, RF is followed by TO or GU.

**Table 8:** Motivation Function.

Teacher	Criteria Conduct	Consequent Conducts			
		Lag 1		Lag 2	
		Codes	Yule’s Q	Codes	Yule’s Q
Therapeutic Pedagogy	RF	TE (5.88)	0.66	AT (8.13)	0.56
Preschool		VR (7.85)	0.56		
Specialized Unit (*Aula Enclave*)		TO (2.55)	0.35	–	
		GU (3.63)	0.44		

*RF, Reinforcement; TE, Teacher’s Explanation; VR, Verbal Review; TO, Teacher’s Organization; GU, Guidance; AT, Answer the Teacher*.

When the criterion behavior is GU, a general pattern occurs in three cases. In the first lag, this behavior is followed by positive interventions by the student, SP and AT. In the second lag teachers continue to guide students in their learning, GU. Also, in the case of TP with students of Preschool Education, in the second lag, GU is followed by NR and in Specialized Unit by RF (**Table 9**).

**Table 9:** Guidance and Advice Function.

Teacher	Criteria Conduct	Consequent Conducts			
		Lag 1		Lag 2	
		Codes	Yule’s Q	Codes	Yule’s Q
Therapeutic Pedagogy	GU	TE (3.19)	0.42	**GU** (10.07)	0.60
Preschool		**SP** (8.70)	0.66	**NR** (3.89)	0.52
		**AT** (7.05)	0.43		
Therapeutic Pedagogy Primary		**SP** (2.80)	0.40	**GU** (4.24)	0.54
		**AT** (4.23)	0.49		
Specialized Unit (*Aula Enclave*)		**SP** (2.95)	0.38	**RF** (4.82)	0.54
		**AT** (7.85)	0.71	**GU** (4.35)	0.45
Therapeutic Pedagogy	NR	**RF** (4.11)	0.57	–	–
Preschool		**GU** (3.48)	0.48		

*TE, Teacher’s Explanation; SP, Student Participation; AT, Answer the Teacher; RF, Reinforcement; GU, Guidance; NR, Non-verbal Revision*.

On the other hand, when the criterion behavior is NR, significant patterns only occur in TP with students of Preschool Education in the first lag. The teacher applies a positive feedback (RF) or guides the task to verify if it is not being done well (GU). These behavioral patterns correspond to good teaching practices (**Table 9**).

The patterns obtained when the criterion behavior is VR are presented in **Table 10**. In this case a general pattern occurs in the three cases in the first lag. The criterion behavior is followed by AT.

**Table 10:** Evaluation Function.

Teacher	Criteria Conduct	Consequent Conducts			
		Lag 1		Lag 2	
		Codes	Yule’s Q	Codes	Yule’s Q
Therapeutic Pedagogy	VR	**AT** (27.37)	0.96	**RF** (14.97)	0.81
Preschool				**VR** (6.85)	0.43
Therapeutic Pedagogy		**AT** (16.00)	0.97	**GU** (3.54)	0.48
Primary				**VR** (7.46)	0.74
Specialized Unit (*Aula Enclave*)		AT (7.11)	0.89	–	–

*AT, Answer the Teacher; RF, Reinforcement; VR, Verbal Review; GU, Guidance*.

In the second lag, in the context of TP with students of Preschool Education and Primary School, the teacher continues VR, monitoring the correct assimilation of the contents they are working.

It is also important to note that teacher continues to apply a positive feedback to the student’s response of Preschool Education, RF, while with students of Primary School, the teacher continues to guide the students, GU.

When the criteria are the positive interventions of students (see **Table 11**) significant patterns are observed in all three cases. In the first lag, SP is followed either by TO or by GU. The latter behavioral pattern is a pattern of good teaching practices.

**Table 11:** Positive Interventions of Students.

Teacher	Criteria Conduct	Consequent Conducts			
		Lag 1		Lag 2	
		Codes	Yule’s Q	Codes	Yule’s Q
Therapeutic Pedagogy	SP	**GU** (14.60)	0.85	TE (4.18)	0.58
Preschool				SP (10.96)	0.79
Therapeutic Pedagogy		TO (8.07)	0.75	SP (2.57)	0.38
Primary		**RF** (4.36)	0.68		
		**GU** (5.48)	0.63		
Specialized Unit (*Aula Enclave*)		TO (4.78)	0.56	–	–

Therapeutic Pedagogy	AT	**RF** (17.90)	0.91	TE (2.32)	0.30
Preschool		**GU** (6.55)	0.40	**VR** (2.47)	0.16
		**NR** (2.87)	0.40	AT (9.72)	0.51
		**VR** (4.32)	0.27		
Therapeutic Pedagogy		**RF** (3.39)	0.45	AT (7.85)	0.67
Primary		**GU** (6.11)	0.63		
		**VR** (5.29)	0.58		
Specialized Unit (*Aula Enclave*)		**RF** (8.89)	0.79	–	–
		**GU** (2.56)	0.31		

*GU, Guidance; TO, Teacher’s Organization; RF, Reinforcement; GU, Guía; NR, Non-verbal Revision; VR, Verbal Review; TE, Teacher’s Explanation; SP, Student Participation; AT, Answer the Teacher*.

Interventions of the students on their own initiative are followed by RF only by the teacher of TP with students of Primary School. This behavioral pattern is also a good teaching practice. The code SP includes those interventions of students that are made on their own initiative, without distinguishing them by their content. This code includes interventions related to curriculum content or other related to organizational aspects. In the case of students of TP of Primary School it is more likely to perform interventions related to the contents they are working, because they cover more difficult content and they are older.

On the other hand, when the criterion behavior is AT, it is followed in all cases by RF, or GU in the first lag. GU and RF are necessary behaviors so that students can progress in their learning. Therefore, these behaviors are good teaching practices.

Also, in TP with both students of Preschool Education and students of Primary School AT is followed by *Review Verbal* (RV) and in the case of Therapeutic Pedagogy with students of Preschool Education in particular, it is followed by NR. These behavioral patterns are also patterns of good teaching practices. In the second lag, in TP with students of Preschool Education, it is followed by either TE or VR. The latter pattern is suitable as the teacher continues to check the proper assimilation of the contents.

Regarding the negative behaviors of students, when the criterion behavior is CD significant patterns are found in Therapeutic Education with students of Primary School. In this context, in the first lag, CD is followed by CL. This behavioral pattern is a good teaching practice (**Table 12**).

**Table 12:** Negative Behaviors of Students.

Teacher	Criteria Conduct	Consequent Conducts			
		Lag 1		Lag 2	
		Codes	Yule’s Q	Codes	Yule’s Q
Therapeutic Pedagogy Primary	CD	**CL** (19.31)	1.00	–	–

*CD, Classroom Disruption; CL, Control*.

Finally, conversations about issues not related to the content given in class are also had in the classroom. These conversations are collected with the GI code. The goal of these interactions is to create a safe and reliable climate, stimulate or generate a contrast that allows students to relax and regain the level of attention when the teacher returns to class. This type of behavior is more expected in TP. Here, the curriculum addressed requires moments of relaxation to allow the students to maintain the appropriate level of attention. However, in Specialized Unit, by the characteristics of the students, they work basic and everyday contents which are approached from a ludic point of view. Therefore, the GI fails to meet its goal of stimulating contrast to become curricular content.

Significant behavioral patterns of GI by the teacher are presented in **Table 13**. In the first lag, these interactions have a response by the students in TP with both students from Preschool Education and Primary School. In the second lag, the teacher continued the conversation again with GI in response to the interventions of students.

**Table 13:** Interaction Function.

Teacher	Criteria Conduct	Consequent Conducts			
		Lag 1		Lag 2	
		Codes	Yule’s Q	Codes	Yule’s Q
Therapeutic Pedagogy	TGI	SGI		TGI	
Preschool		(30.46)	0.99	(22.62)	0.97
Therapeutic Pedagogy		SGI		TGI	
Primary		(16.65)	0.98	(11.88)	0.94

*SGI, General Interactions by the Students; TGI, General Interactions by the Teacher*.

Significant behavioral patterns of GI by the students are presented in **Table 14**. In the first lag, these interactions are answered by the teacher and, in the second lag, the conversation has continued again with GI of students.

**Table 14:** Neutral Interventions of Students.

Teacher	Criteria Conduct	Consequent Conducts			
		Lag 1		Lag 2	
		Codes	Yule’s Q	Codes	Yule’s Q
Therapeutic Pedagogy	SGI	TGI		SGI	
Preschool		(29.75)	0.99	(24.09)	0.98
Therapeutic Pedagogy		TGI		SGI	
Primary		(18.06)	0.99	(13.24)	0.95

*TGI, General Interactions by the Teacher; SGI, General Interactions by the Students*.

These conversations are good teaching practices when their aim is to create a safe and trustworthy climate for the students or to create a stimulating contrast.

## Discussion

The challenge of education is to offer it in terms of quality and equity for all. One of the important aspects of this is to address and respond to the different educational needs presented by students. This requires assessing, among other things, the behavior of teachers in their professional performance in the classroom, as they are responsible to teach and make students learn (Hernández, 2006). Specifically, teachers dedicated to Special Education have special relevance in attention to this diversity in the classroom.

As we have seen in this work, the observational methodology offers us a lot of information but it is important to apply it properly. It is decisive to use valid, accurate, reliable observation instruments. In addition, due to the cost to the time and resources that can be assumed by this methodology, we need to have procedures which allow us to apply it efficiently. In this work we have begun using mathematical procedures as optimization GT (Blanco-Villaseñor, 1991; Blanco and Anguera, 2000; Blanco et al., 2000, 2010) to establish the minimum of codifying time necessary to obtain accurate information that allows its generalizability. From the results obtained in the optimization, we include new sessions. Thus was to ensure the stability of the conduct of the teachers we studied (Borges and Rodríguez-Dorta, 2015; Rodríguez-Dorta and Borges, 2015b).

The behavioral analysis of the teachers from this study allows us to see that they adapt their behavior according to the students with whom they work, giving the most adequate response to their needs.

As it was mentioned, the teacher of TP works with both students from Preschool and Primary Education. The results obtained indicate his behavioral patterns changes depending on the students with who he is working at every moment. Therefore he adapts his way of teaching according to the needs and characteristics of his students. The teacher from Specialized Unit also shows different behavioral patterns.

In the case of the teacher from the Specialized Unit and one of TP when he works with children from Primary School, there are significant patterns in TO follow by the response of the students, AT, and again TO. In addition, in the case of TP with students of Primary School to TO is followed, in the first lag, TE, which is logical since the contents of this educational stage require theoretical explanations.

*Non-verbal Revision* produces significant patterns only in the case of the TP teacher when teaching classes to students of Preschool Education. Thus, after the NR applies RF to the student or continues to guide him on his learning (GU). On the other hand, the student’s response to questions or approaches of the teacher receives non-verbal supervision (NR). Since there is only one student from this educational stage, the supervision is more intensive and continuous and the teacher is totally focused on him.

The code VR also produces a general pattern in all the cases (VR–AT). However, it is in the case of the teacher of TP where the teacher continues checking to if the students are understanding what they are working on after a response from the students (AT) or a theoretical explanation (TE). This verification process is of great importance in TP. However, in Specialized Unit the priority is another. This discipline supposes reinforcement for students with special educational needs whose objective is to get them to acquire the same knowledge as their regular classmates. However, in Specialized Unit, more than the verification of the acquisition of content, what is really important is to motivate and to guide the students to perform their tasks. Here, due to the educational needs presented by the students, the objective is they become more independent and they again autonomy, moving away from the contents raised with their regular classmates.

Regarding the positive interventions of the students, the answers of the students (AT) to questions and approaches of the teacher, receive both RF and GU by the teacher in all the cases. However, the SP on its own initiative only receives GU in TP with students from Preschool Education and Primary School. RF is obtained in TP with students from Primary School in particular. It is possible that the interventions on the student’s own initiative refers to what they are doing, but on the other two cases, due to the age and characteristics of the students, they refer more to aspects of organizational and general character.

It is important to note how both teachers only use reinforcement to generate motivation. However, there are no behavioral patterns where the teacher encourages the students to the task by highlighting some positive aspect of it, show anticipation of reward or encourage the students to participate. Aspects collected in the instrument of observation through the code MO.

With regard to the negative interventions of students, CD, only produce a significant pattern in TP with students of Primary School. This behavior is followed adequately by CL. This is logical because the teacher of TP teaches three students of Primary School, while Preschool Education only teaches a student. With a greater number of students the teacher requires a greater control and regulation of the group.

The code GI produces a significant reciprocal pattern in the two cases of TP (with students of Preschool Education and with students of Primary School) but not in Specialized Unit. These interactions, as discussed above, are important insofar as these interactions are intended to create a safe and trusting environment and to be a contrast encouraging allowing the students to rest and return to maintain the attention once the task is resumed. In Specialized Unit the contents are very practical. In this case these topics become part of the curriculum to be taught.

To the extent that the patterns developed by the teachers studied are adapted to the different needs presented by the students are indicative of good teaching practices. The patterns obtained indicate that the teachers observed offer support, help, and clear guidelines to perform the tasks through GU after interventions of the students (SP and AT) or after non-verbal supervision of their work (NR) (SP–GU, AT–GU, and NR–GU). This is in line with the results of the research that points out, as a relevant aspect for effective teaching, the importance of the teacher solving the possible doubts that arise in students (Berliner, 1983; Anderson, 1989, 2004) through support (“Scaffolds”) to students so that they can carry out the activities (Palincsar and Brown, 1984; Van de Grift, 2007) and among other things, presenting new material in small steps, providing clear and detailed instructions, asking questions and providing guided practice (Rosenshine, 1979; Muijs and Reynolds, 2003; Houtveen et al., 2004; Houtveen and Van de Grift, 2006).

Another aspect to mention and which is indicator of good practices is they motivate their students to learn by giving them positive feedback (RF) after student interventions or non-verbal supervision of their work (NR–RF, SP–RF, and AT–RF). Several researches highlight the motivation of students as an important aspect for effective teaching. Thus, research refers, among other aspects, to strategies such as individualized attention, reinforcement, public and private praises, etc. (Murillo et al., 2011).

The teachers observed check their students assimilated the contents using questions or non-verbal supervision (VR and NR) after a theoretical explanation (TE) or a response of the students (AT) (TE–VR and AT–VR). The evaluation and the continuous monitoring of students have also been referred by researchers as an aspect which favors effective teaching. These aspects not only allow evaluating the achievements reached by the students, but also provide a motivation strategy for them by offering feedback on their learning process (Muijs and Reynolds, 2001; Anderson, 2004; Hattie and Timperley, 2007; Murillo et al., 2011; Pegalajar-Palomino, 2011). Specifically, literature refers to frequent supervision (Daloz, 1986; Stronge et al., 2004; Killen, 2005; Brookhart, 2009; Orlich et al., 2010; Murillo et al., 2011), asking questions or observing the work that students are doing (Pellicer and Anderson, 1995).

Another important issue on this topic is related with the discipline on the classroom. Thus, an indicative pattern of good practices in the teachers studied is that after CD the teacher applies CL. Applying an adequate control method to an inappropriate behavior allows the students to obtain the maximum benefit from teaching (Omoteso and Semudara, 2011).

Finally, these teachers try to generate a safe climate through GI and, at the same time, with this type of interactions they facilitate the students moments of disconnection that allows them to return to the task with a higher level of attention. Students should feel safe and comfortable to participate in the activities (Muijs and Reynolds, 2001). Literature refers to the climate that is generated in class as one important aspect which has to be taken into account for effective teaching. Thus, simple questions such as greeting or asking students for general aspects favor an adequate climate for learning (Hernández-Jorge, 2005).

These results show that the observational instrument used in this research, PROFUNDO-EPE, v.3, allows to capture the relevant aspects of Special Education professionals through a dynamic evaluation of teacher performance in the classroom. Thus, it is useful to evaluate the initial and permanent education of these teachers, allowing the detection of possible difficulties and establishing recommendations for improvement.

The limitation of this work is that it has been carried out only with two teachers. To check whether the conduct of special education teachers is different depending on the context and if it is appropriate, it is necessary to extend this study to a larger number of teachers in different contexts of Special Education.

Additionally, it is important to point out that the observational instrument used on this study shows, through the Teaching Functions, *what* the teachers does in their educational work in the classroom. However, it would be extremely interesting to be able to capture *how* they do it, that is, the specific strategies that the teacher use to explain, guide, reinforce or evaluate. These aspects have begun to be analyzed with university teacher through the Protocol of Observation of the Function of Explanation (PROFE in Spanish) (Borges et al., 2016b). This protocol has been designed to operationalize how the teacher transmits knowledge, collecting all those strategies, resources or observable styles that the teacher uses during the theoretical exposition of the contents.

This aspect has great relevance in order to capture behavioral patterns which represent good teaching practice. The research about teachers and teaching effectiveness refers to specific strategies used by teachers within each of the teaching functions.

Undoubtedly, a particularly important aspect for achieving quality education is that it is capable of giving an appropriate answer to the different educational needs presented by students. Thus, education could maximize their abilities and ensure adequate personal and social development. Therefore, the efforts directed to assess that this is being achieved are a requirement on this area. This requires an evaluation on the development of the educational process that is formative evaluation (Tejada, 1999; López de la Llave and Pérez-Llantada, 2004). It is very important to provide quality education.

## Ethics Statement

The results of this study are part of the research from a doctoral thesis (“*Evaluation of the process of teaching behavior in Education Primary and Special*”) that was done and presented in the University of La Laguna. This doctoral thesis has the ethical approval of the Ethics Committee of Research and Animal Welfare (CEIBA, in Spanish) of the University of La Laguna, with registration number CEIBA2017-0224. In accordance with the Organic Law 15/1999 of December of Protection of Personal Data (1999, BOE n° 298 of December 14) the written informed consent was obtained from all individual participants included in the study (teachers and parents), where they agreed to participate in the investigation, as well as recording their behavior.

In addition, and following the guidelines of the aforementioned law, we request the written observer’s confidentiality agreement.

## Author Contributions

MR-D has participated in the entire study. AB has also participated in the totality of the work (theoretical review, study planning, method and discussion), except in the analysis. Both authors have participated in the writing of the article.

## Conflict of Interest Statement

The authors declare that the research was conducted in the absence of any commercial or financial relationships that could be construed as a potential conflict of interest.
